# Supplementary material for: Ionic transport through sub-10 nm diameter hydrophobic high-aspect ratio nanopores: experiment, theory and simulation
Source: Sci Rep. 2015 Jun 3;5:10135. doi: 10.1038/srep10135 (PMC4453161; doi:10.1038/srep10135)
Supplement: Supplementary Information [file srep10135-s1.pdf]

Ionic transport through sub-10 nm diameter hydrophobic high aspect ratio nanopores: experiment, theory and simulation

Sébastien Balme[1]‡*, Fabien Picaud[2]‡, Manoel Manghi[3]‡, John Palmeri[4]‡, Mikhael Bechelany[1], Simon Cabello-Aguilar[1], Adib Abou-Chaaya[1], Philippe Miele[1], Emmanuel Balanzat[5], Jean Marc Janot[1]

Nanopore characterization

SAXS Characterization: In order to determine the growth per bilayer Al_2O_3 / ZnO nanolaminates, SAXS was performed on 35 sequences of 5 cycles Al_2O_3 / 5 cycles ZnO deposited on PC track etched membrane with a pore diameter of 200 nm. The SAXS profile shows 2 main features: (i) a structure peak at q = 2.53 nm^{-1}, corresponding to a characteristic distance of 2.48 nm in the real space, this distance corresponds to the width of a double-layer deposited by ALD and (ii) A q^{-4} slope in the low q region, characteristic of a sharp interface between the air in pores and the layers deposited by ALD. This is a proof of the quality of the deposition in terms of width control and homogeneity.

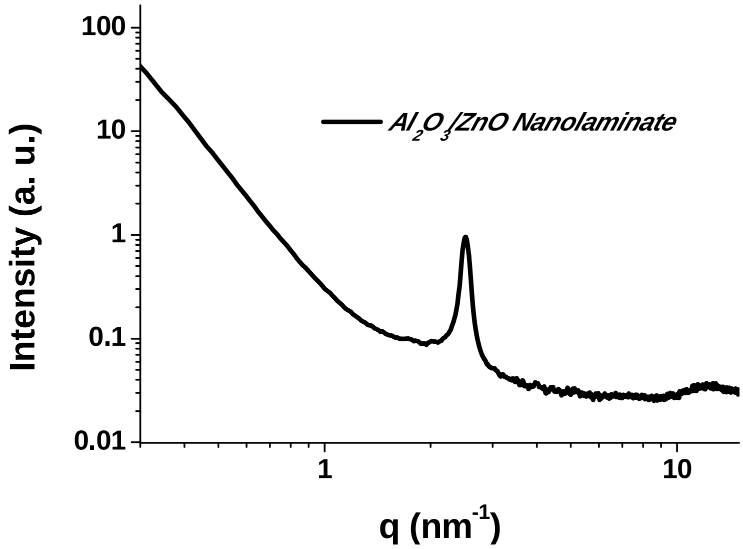

Figure SI-1. SAXS profile obtained on PC track etched membrane (Whatman-Nucleopore, diameter 200 nm, density 7.10^8 pore cm^{-2}) after 35 sequences of 5 cycles Al_2O_3 / 5 cycles ZnO

XPS Characterization: XPS measurement was performed on PET membrane with 3 sequences of 5 cycles Al_2O_3 / 5 cycles ZnO before and after HMDS modification (Table SI1) in order to confirm the TMS grafting on the surface. The success of the grafting has been attested by the observation of Silicon in low content (Si 2p binding energies 100.38 eV) that corresponds to Si-CH_3 bond. In addition contact angles with water were measured by applying a water droplet of 2.3 μL to the surface. The hydrophobicity was confirmed by the determination of a contact angle of 92°C on the treated HMDS surface.

	**PET/Al_2O_3/ZnO**		**PET/Al_2O_3/ZnO/HMDS**	
	Peak BE (eV)	***At. %***	***Peak BE (eV)***	***At. %***
**Al2p**	73.83	11.96	74.01	11.46
**C1s**	284.82	21.40	284.78	25.82
**O1s**	531.32	46.76	531.50	43.48
**Zn2p3**	1021.56	19.88	1021.66	17.89
**Si2p**	-	-	100.39	1.34

Table SI-1. XPS measurement on nanopores before and after HMDS grafting

MD simulations

	NT2 *D*=2nm, *L*=5nm			NT3 *D*=3nm, *L*=5nm			NT5 *D*=5nm, *L*=5nm			NT6 *D*=6nm, *L*=3nm		
Neopentane number	96			128			195			150		
Concentration	0.1M	0.5M	1M	0.1M	0.5M	1M	0.05M	0.5M	1M	0.1M	0.5M	1M
H_2O number	4616	4548	4382	9203	9065	8891	27515	27118	26535	9544	9530	9512
Na^+ number	9	43	86	17	86	173	26	258	516	2	9	18
Cl^- number	9	43	86	17	86	173	26	258	516	2	9	18

Table SI-2: Molecular and ionic composition of the simulated systems.

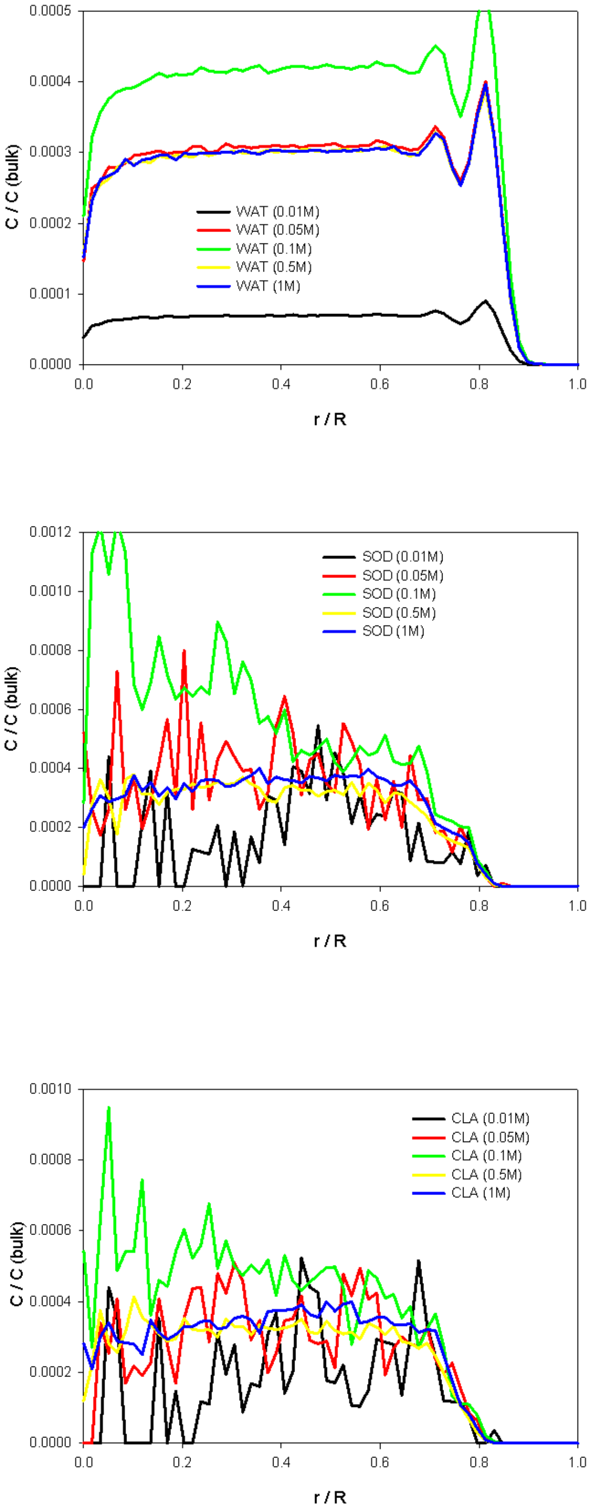

Figure SI-2: Concentration profiles of water (top) and sodium Na^+ (middle) and chloride Cl^-(bottom) ions, normalized by their bulk concentrations as a function of *r/R*, for ionic bulk concentrations c_s = 0.01, 0.05, 0.1, 0.5, and 1M and *R* = 3nm.

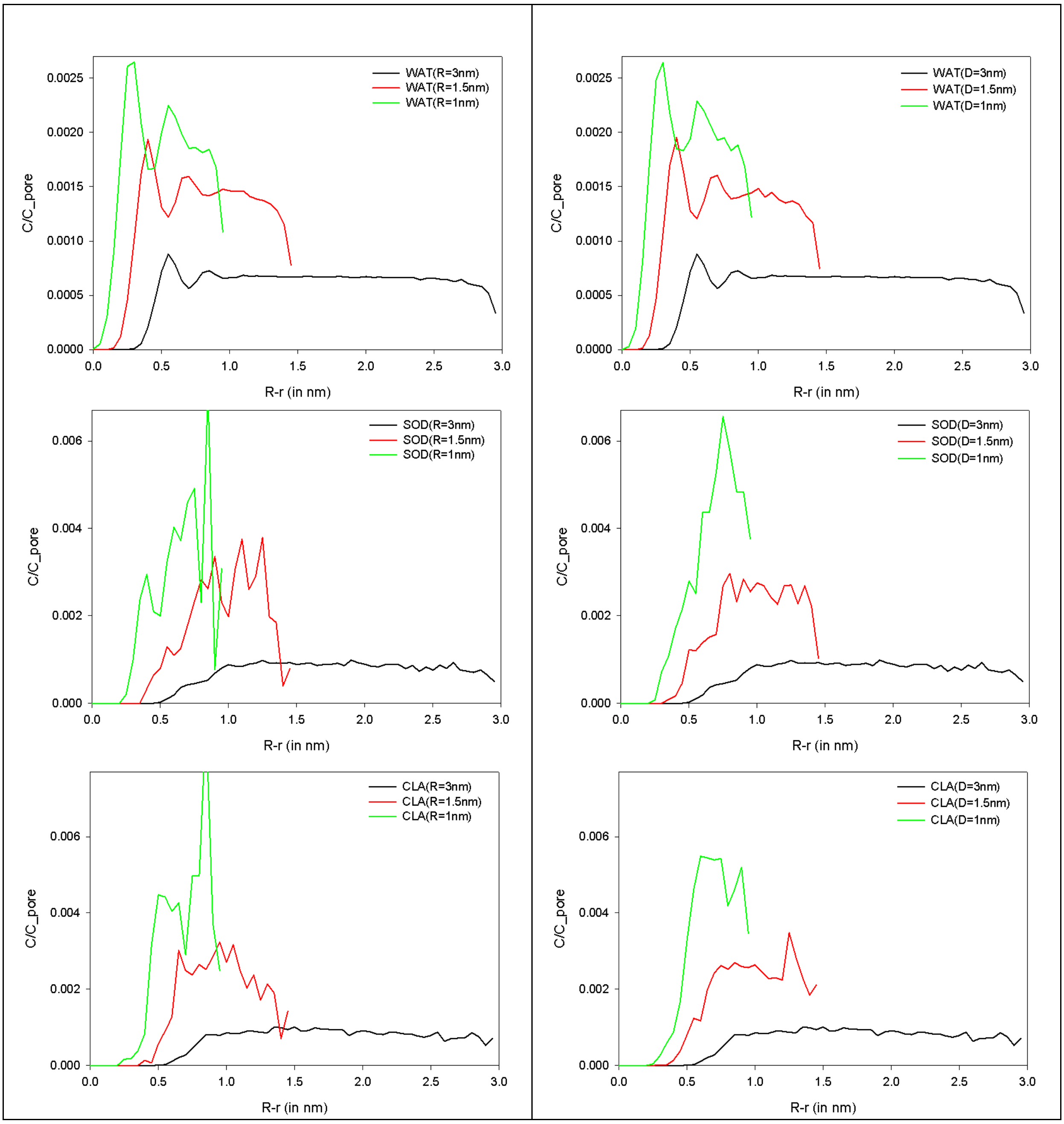

Figure SI-3: Concentration of the different species with respect to their pore concentration as a function of the distance from the pore wall [ionic bulk concentrations equal to 0.1M (left panel) or to 1M (right panel)]

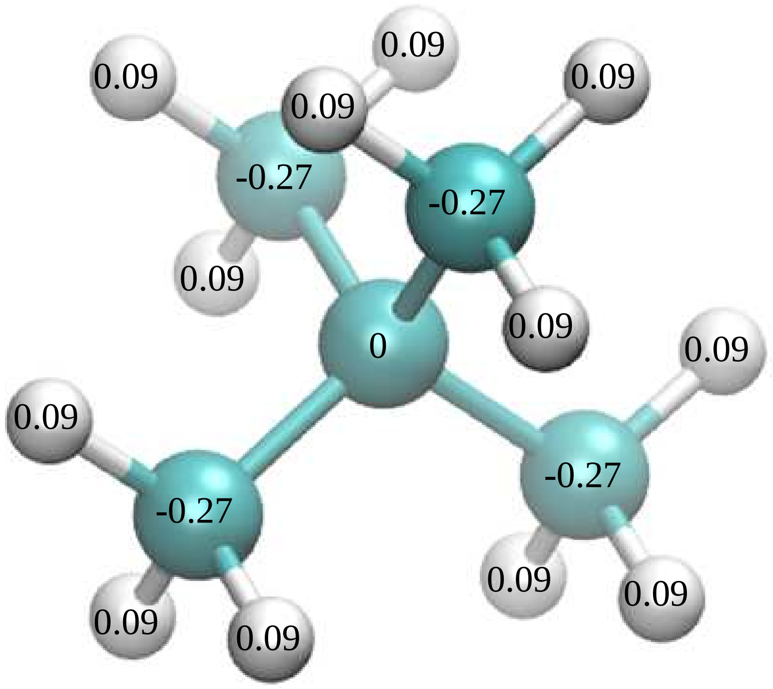

Figure SI-4: Geometry of the neopentane molecule and charge repartition

Derivation of the nanopore conductivity

Space charge (or PNP) model

To gain further physical insight into the nature of ionic conductivity in charged nanopores we rederive the conductivity and show how to obtain the limiting form in the homogeneous approximation. The pore level PNP approach, also known as the *space charge model*, to ion transport in charged cylindrical pores, takes into account only the electrostatic interactions. It is described by the following set of equations: the molar flux densities $\mathbf{j_i}$ in the form of the extended Nernst-Planck (ENP) equations, incorporating ionic diffusion, electrical migration, and convection (for each ion *i),*

$$\mathbf{j}_i = -D_i \nabla c_i - z_i e \mu_i c_i \nabla \psi + c_i \mathbf{v}, \qquad \text{equation A1}$$

where $\mu_i = D_i / (k_B T)$ is the ion mobility, D_i the diffusion coefficient, c_i the local concentration, z_i the valence, ψ the local electric potential, and $\mathbf{v}$ the solution flow velocity. The flow velocity obeys the (creeping flow) Stokes equation:

$$-\nabla p + \eta \nabla^2 \mathbf{v} - \rho_c \nabla \psi = 0 \qquad \text{equation A2}$$

with p the local pressure, η the solution viscosity, $\rho_c = e\sum_{i=1}^{N} z_i c_i$ the ionic charge density, and N the total number of ions. The electric current density is given by

$$\mathbf{j}_c = e\sum_{i=1}^{N} z_i \mathbf{j}_i \qquad \text{equation A3}$$

The local electric potential is governed by the Poisson equation:

$$\nabla^2 \psi = -\frac{\rho_c}{\varepsilon} = -\frac{e}{\varepsilon}\sum_{i=1}^{N} z_i c_i \,, \qquad \text{equation A4}$$

where ε is the dielectric constant of the solution. The boundary conditions on the radial ENP equations are

$$\frac{\partial c_i}{\partial r}\Big|_{r=r_p} + c_i \frac{z_i e}{k_B T}\frac{\partial \psi}{\partial r}\Big|_{r=R} = 0 \quad \text{(no radial flux at pore wall)} \qquad \text{equation A5}$$

and by symmetry, $\frac{\partial c_i}{\partial r}\Big|_{r=0} = 0$ and $\frac{\partial \psi}{\partial r}\Big|_{r=0} = 0$. The pore wall surface charge density, σ, is related to the potential gradient at the pore wall by Gauss' law:

$$\frac{\partial \psi}{\partial r}\Big|_{r=R} = \frac{\sigma}{\varepsilon}\,, \qquad \text{equation A6}$$

which can also be put into dimensionless form, $\partial\tilde{\Psi} / \partial\tilde{r}\Big|_{\tilde{r}=1} = \sigma^*$, where $\tilde{\Psi} = -ez_i\psi / (k_B T)$ $\tilde{r} = r / R$ and σ^* is the dimensionless surface charge density (Eq. (5) in the main text).

Homogeneous approximation

When $\sigma^* < 1$, radial variations are weak enough for the homogeneous approximation to be valid. (The validity of the GCE approximation is delimited by $-e\beta\phi_{GCE}$ (0) > 1, which shows that this approximation holds only at sufficiently low salt concentration or sufficiently small pore diameter via the ratio R/λ_{DH}, with the threshold value depending on the surface charge density.) In this approximation the space charge model simplifies to the following set of equations (planar symmetry, with physical quantities varying only in the axial *x* direction): the spatially averaged ion flux density,

$$J_i = -D_i \partial_x \bar{c}_i - ez_i \bar{c}_i \mu_i \partial_x \phi + \bar{c}_i J_v \qquad \text{equation A7.}$$

(As is usually done, we have neglected a small term proportional to the pressure gradient and the specific ion volume.) The averaged (creeping flow) Stokes equation for the flux of a charged fluid takes the form

$$\frac{1}{L_p^0} J_v = -\partial_x P - \rho_c \partial_x \phi, \qquad \text{equation A8}$$

with $0 \le x \le L$ (membrane thickness or nanopore length), where $\rho_c = e\sum_i z_i \bar{c}_i = -eX_m$ is the local ion charge density, $X_m = -\sum_i z_i \bar{c}_i = 2\sigma/(eR)$ is the average concentration of pore charge, and $J_v \approx \langle v_x \rangle$ is the volume flux density (approximately equal to the average solution velocity). The radial PB equation simply reduces to pore averaged electroneutrality in the homogeneous limit. The second term in the Stokes equation A8 arises from the electric body force acting on a charged liquid. In the homogeneous theory we use the following quantities: $\bar{c}_i = \langle c_i \rangle$: average concentration of ion *i* in the membrane; $\phi = \langle \psi \rangle$: average intra-membrane electric potential; $P = \langle p \rangle$: average pressure ; and L_p^0: pure water hydraulic permeability.

In general there will be ionic concentration, pressure, and electric (Donnan) potential jumps at the interfaces separating the external reservoirs from the pore entrances. In the calculation of the pore conductivity there is an applied electric field and corresponding potential difference $\Delta\phi$ across the nanopore. In this situation the ionic concentrations are the same on both sides of the pore and therefore the pressure, and electric (Donnan) potential jumps cancel. The concentration jumps are embodied in the ionic partition coefficients, $k_i = \bar{c}_i / c_i$ where $c_i = \nu_i C$ and C is the external salt concentration and ν_i the *stoichiometric* coefficient. The ionic electric current density, $J_c = e\sum_{i=1}^{N} z_i J_i$, can then easily be calculated in the absence of concentration and pressure differences from Eqs.A7-8, leading to

$$J_c = -e^2\left(\sum_{i=1}^{N} z_i^2 \bar{c}_i \mu_i\right)\Delta\phi - eX_m J_v \qquad \text{equation A9}$$

with

$$J_v = -L_p^0 \rho \Delta\phi = L_p^0 e X_m \Delta\phi . \qquad \text{equation A10}$$

The first term in equation A9 arises from electrical migration of ions in the applied electric field and the second (electro-osmotic) term arises from the advective ionic current density created by the solution flow induced by an electric field, even in the absence of a pressure gradient. The conductivity in the homogeneous approximation is then given by

$$\kappa_{h,slip} = -\frac{J_c}{\Delta\phi} = e^2\sum_{i=1}^{N} z_i^2 \bar{c}_i \mu_i + \frac{4\sigma^2}{R^2} L_p^0 . \qquad \text{equation A11}$$

In the presence of flow slip at the pore wall, the pure water hydraulic permeability is given by

$$L_p^0 = \frac{R^2}{8\eta}\left(1+\frac{4b}{R}\right), \qquad \text{equation A12}$$

where b is the slip length. We then find

$$\kappa_{h,slip} = e^2 \nu_i |z_i| C \sum_{i=1}^{N} |z_i| \mu_i k_i + \frac{\sigma^2}{2\eta}\left(1+\frac{4b}{R}\right). \qquad \text{equation A13}$$

The slip correction,

$$\delta\kappa_{slip} = \frac{2\sigma^2 b}{\eta R}, \qquad \text{equation A14}$$

obtained here in the homogeneous approximation, is in fact exact because slip adds a constant component (independent of r) to the flow field and therefore the slip contribution to the conductivity depends only on global electro-neutrality in the pore, and not at all on the radial concentration profile (see below).

General result for the slip contribution to conductivity

In the absence of axial pressure and ion concentration gradients the axial component of the Stokes equation reduces to

$$\eta \nabla_r^2 v_x - \rho_c \partial_x \phi = 0, \qquad \text{equation A15}$$

where $\nabla_r^2 \equiv r^{-1}\partial_r(r\partial_r)$ is the radial part of the Laplacian in cylindrical coordinates. The full electric potential can then be decomposed as

$$\psi(x,r) = \Phi(r) + \phi(x) \qquad \text{equation A16}$$

with $\phi(x) = -xE_0$ arising from the imposed potential difference, $\Delta\phi = -LE_0$ (where L is the length of the nanopore) and $\Phi(r)$ controlling the radial ion distribution via the PBE:

$$\nabla_r^2 \Phi = -\frac{\rho_c}{\varepsilon} = -\frac{eC}{\varepsilon}\sum_{i=1}^{N} z_i \nu_i \exp\left[-z_i e\Phi/(k_B T)\right]. \qquad \text{equation A17}$$

Inserting A17 in A15 yields $\nabla_r^2\left[\eta v_x + \varepsilon\Phi\partial_x\phi\right] = 0$, which can be solved by

$$v_x(r) = -\frac{\varepsilon}{\eta}\partial_x\phi\,\Phi(r) + v_0, \qquad \text{equation A18}$$

where v_0 is a constant fixed by the (slip) boundary condition:

$$\left[v_x(r) + b\partial_r v_x(r)\right]_{r=R} = 0. \qquad \text{equation A19}$$

The slip boundary condition leads, via Gauss' law

$$\left.\frac{\partial \Phi}{\partial r}\right|_R = \frac{\sigma}{\varepsilon}, \qquad \text{equation A20}$$

to

$$v_0 = \frac{\varepsilon}{\eta}\partial_x\phi\,\Phi(R) + \frac{b\sigma}{\eta}\partial_x\phi. \qquad \text{equation A21}$$

The slip contribution to v_0 is $v_{0s} = b\sigma\eta^{-1}\partial_x\phi$, which leads to the slip contribution to the local axial electric current density: $j^s_{x,c}(r) = v_{0s}\rho_c(r)$. The advective contribution to the pore averaged electric current density is then $J_{x,c} = \langle j_{x,c}(r)\rangle = \langle v_x(r)\rho_c(r)\rangle$ and the slip contribution is

$$J^s_{x,c} = \langle j^s_{x,c}(r)\rangle = v_{0s}\langle \rho_c(r)\rangle = -\frac{2b\sigma^2}{\eta R}\partial_x\phi, \qquad \text{equation A22}$$

obtained using $\langle \rho_c\rangle = e\sum_i z_i\langle c_i\rangle = -eX_m = -2\sigma/R$. The conductivity is then $\kappa = -J_{x,c}/\partial_x\phi$ and the non-slip contribution yields the second (advective) term in Eq. 3. Within the scope of the PNP model, the general result for the slip correction to the conductivity is then the same as that previously found using the homogeneous approximation:

$$\delta\kappa_{slip} = -\frac{J^s_{x,c}}{\partial_x\phi} = \frac{2\sigma^2 b}{\eta R}. \qquad \text{equation A23}$$

Slip increases the electro-osmotic contribution and plays an important role in the homogeneous approximation if $b > R/4$: for a 1:1 salt

$$\kappa_{h,slip} = \kappa_h + \delta\kappa_{slip} = e^2C\left(\mu_+k_+ + \mu_-k_-\right) + \frac{\sigma^2}{2\eta}\left(1+\frac{4b}{R}\right). \qquad \text{equation A24}$$

(cf. Eq. 7). In the limit of low salt concentration (GCE regime) $k_+ \approx |X_m|/C$ with $k_- << k_+$ (for a negatively charged pore), which leads in the homogeneous approximation to

$$\kappa^{GCE}_{h,slip} = \kappa^{GCE}_h + \delta\kappa_{slip} = \frac{2e\mu_+|\sigma|}{R} + \frac{\sigma^2}{2\eta}\left(1+\frac{4b}{R}\right). \qquad \text{equation A25}$$

(cf. Eq. 9). The conductance is given by $G = \pi R^2\kappa/L$, therefore a linear increase in G with R at low salt concentration could arise from both the electrical migration term and the slip contribution to the advective part (see Figure 4).
